# Supplementary material for: Optical Transmission in Single-Layer Brain Tissues under Different Optical Source Types: Modelling and Simulation
Source: Bioengineering (Basel). 2024 Sep 13;11(9):916. doi: 10.3390/bioengineering11090916 (PMC11428375; doi:10.3390/bioengineering11090916)
Supplement: Supplementary file 1 [file bioengineering-11-00916-s001.zip › bioengineering-3195201-supplementary.pdf]

bioengineering

MDPI

Supplementary Materials:

Xi Yang [1,2], Chengpeng Chai [1,2], Yun-Hsuan Chen [1,2,*] and Mohamad Sawan [1,2,*]

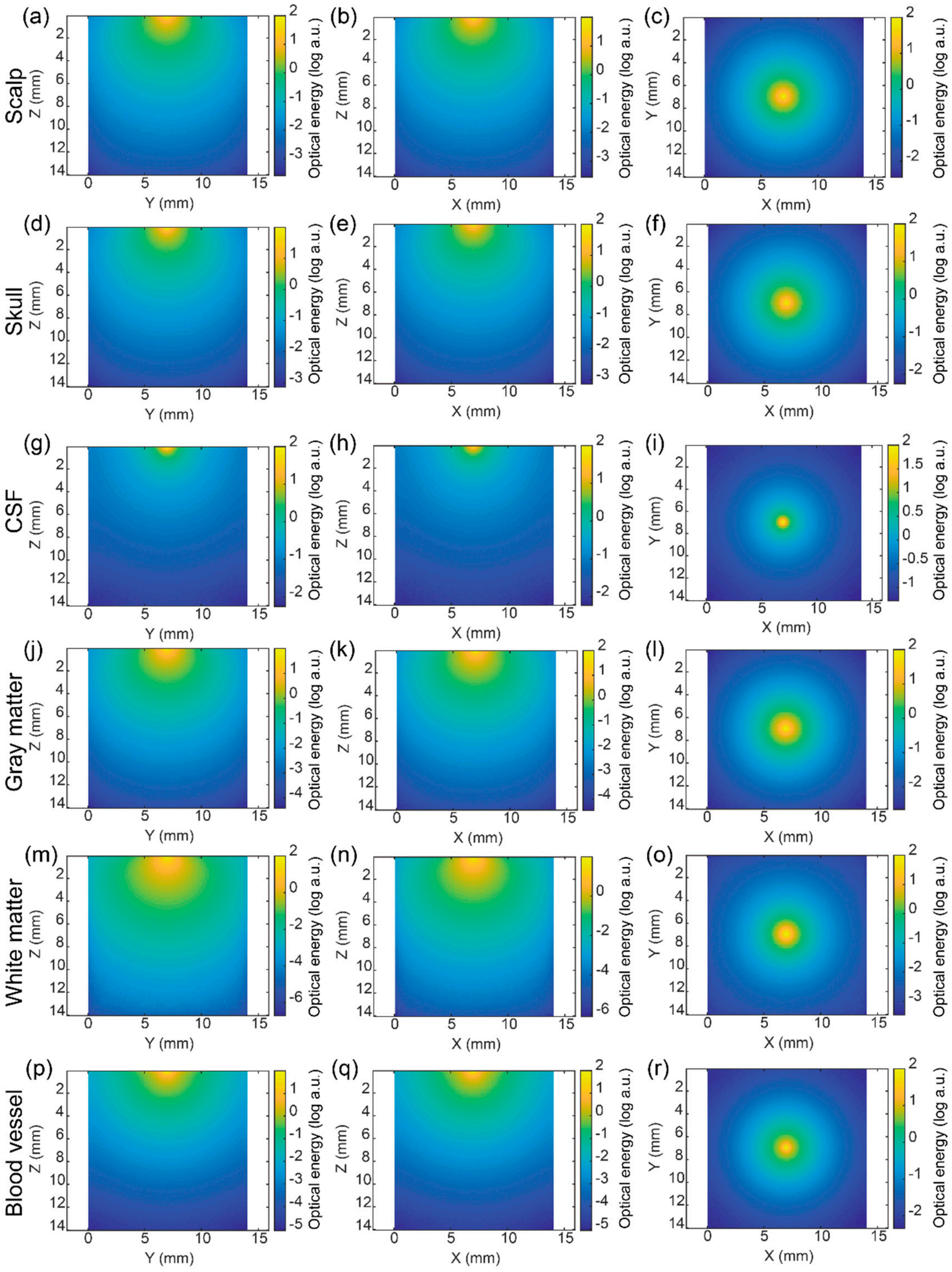

Figure S1. Optical energy distribution of single-layer brain tissues under the illumination of an isotropic source. **(a-c)** Scalp, **(d-f)** skull, **(g-i)** CSF, **(j-l)** gray matter, **(m-o)** white matter, and **(p-r)** blood vessel; **(a, d, g, j, and m)** on the YZ plane (x = 7 mm), **(b, e, h, k, and n)** on the XZ plane (y = 7 mm), **(c, f, i, l, and r)** the XY plane (z = 0.1 mm), respectively.

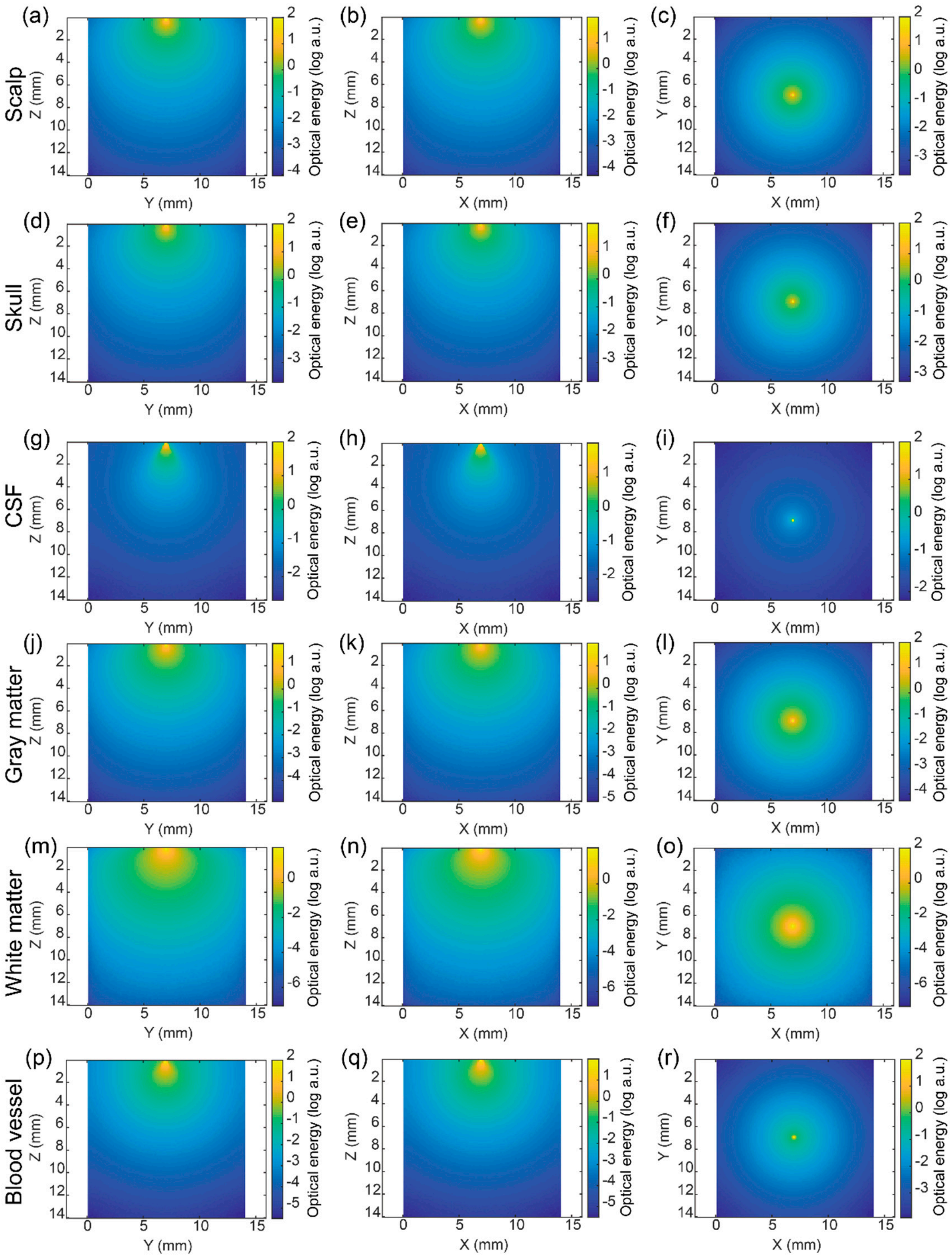

Figure S2. Optical energy distribution of single-layer brain tissues under the illumination of a cone source. **(a-c)** Scalp, **(d-f)** skull, **(g-i)** CSF, **(j-l)** gray matter, **(m-o)** white matter, and **(p-r)** blood vessel; **(a, d, g, j, and m)** on the YZ plane ($x = 7$ mm), **(b, e, h, k, and n)** on the XZ plane ($y = 7$ mm), **(c, f, i, l, and r)** the XY plane ($z = 0.1$ mm), respectively.

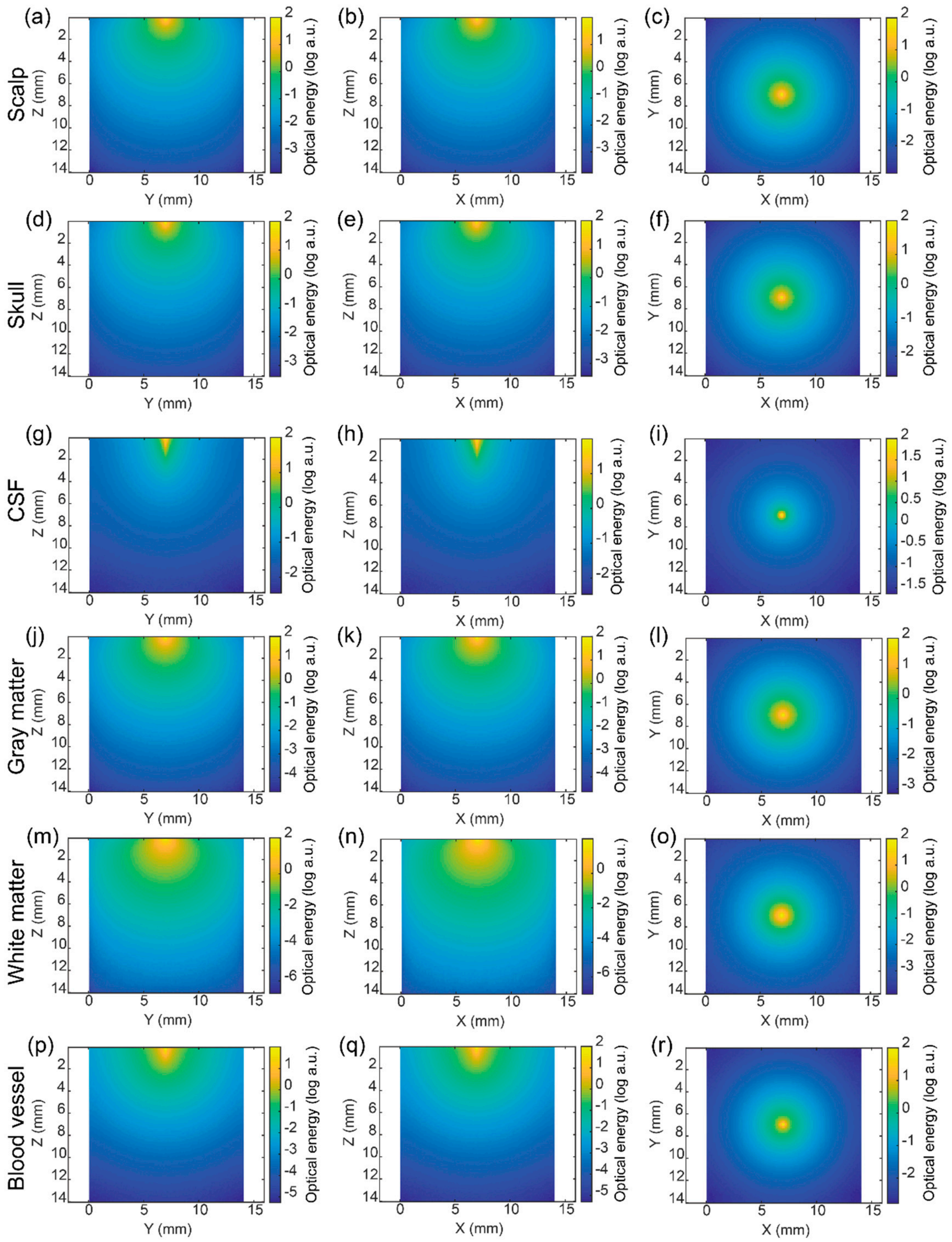

Figure S3. Optical energy distribution of single-layer brain tissues under the illumination of an arcsine source. **(a-c)** Scalp, **(d-f)** skull, **(g-i)** CSF, **(j-l)** gray matter, **(m-o)** white matter, and **(p-r)** blood vessel; **(a, d, g, j, and m)** on the YZ plane (x = 7 mm), **(b, e, h, k, and n)** on the XZ plane (y = 7 mm), **(c, f, i, l, and r)** the XY plane (z = 0.1 mm), respectively.

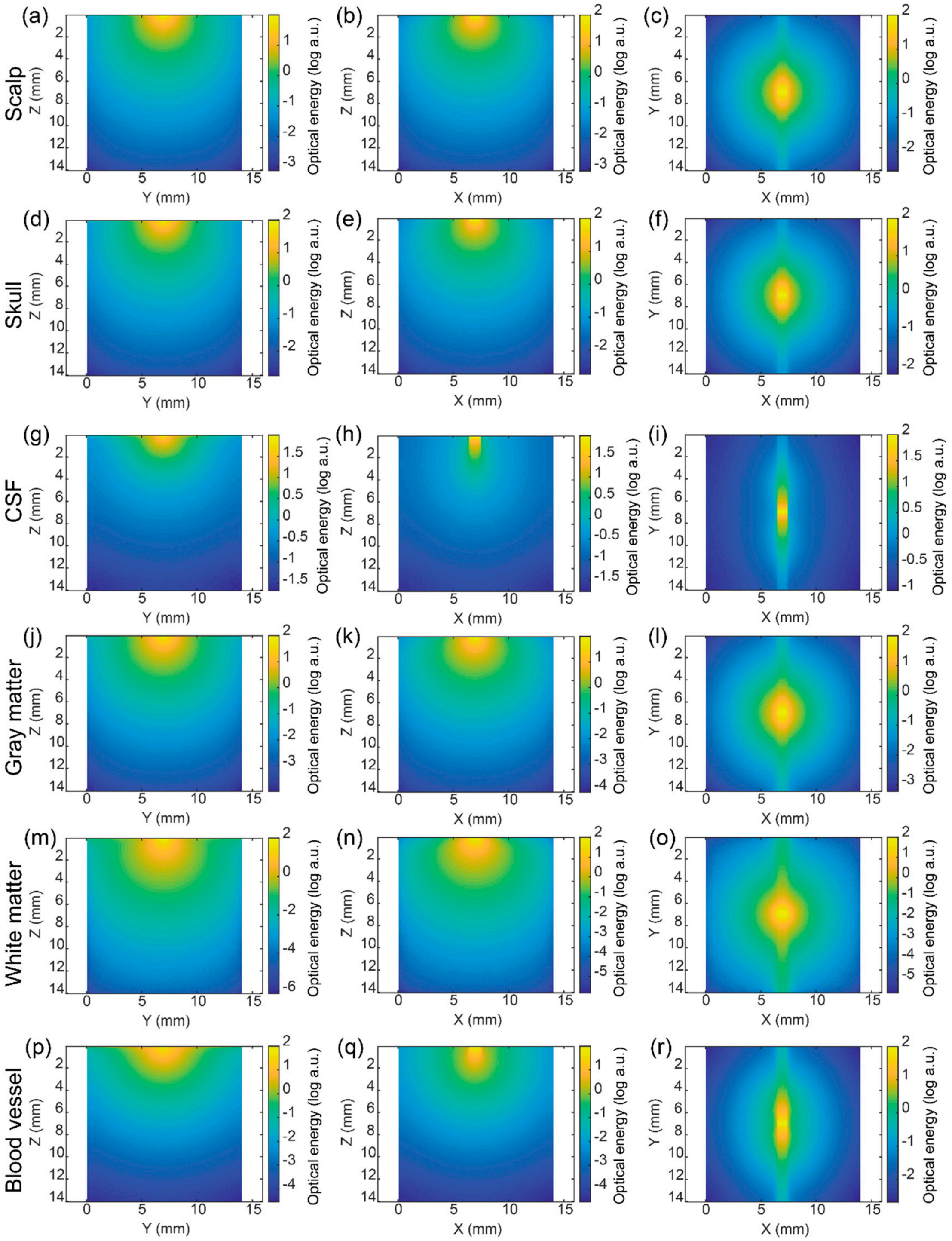

Figure S4. Optical energy distribution of single-layer brain tissues under the illumination of a line source. **(a-c)** Scalp, **(d-f)** skull, **(g-i)** CSF, **(j-l)** gray matter, **(m-o)** white matter, and **(p-r)** blood vessel; **(a, d, g, j, and m)** on the YZ plane (x = 7 mm), **(b, e, h, k, and n)** on the XZ plane (y = 7 mm), **(c, f, i, l, and r)** the XY plane (z = 0.1 mm), respectively.

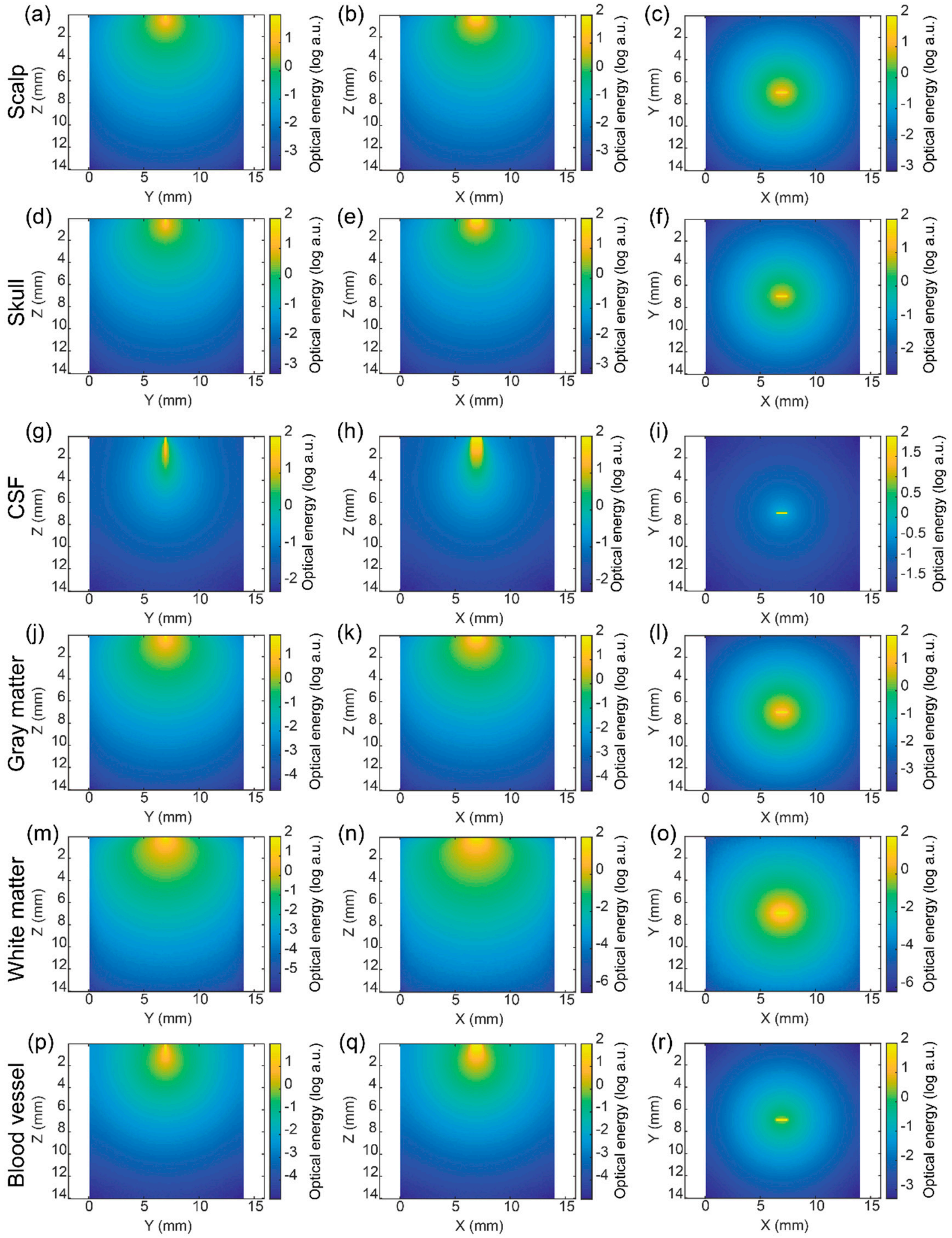

Figure S5. Optical energy distribution of single-layer brain tissues under the illumination of a slit source. **(a-c)** Scalp, **(d-f)** skull, **(g-i)** CSF, **(j-l)** gray matter, **(m-o)** white matter, and **(p-r)** blood vessel; **(a, d, g, j, and m)** on the YZ plane ($x = 7$ mm), **(b, e, h, k, and n)** on the XZ plane ($y = 7$ mm), **(c, f, i, l, and r)** the XY plane ($z = 0.1$ mm), respectively.

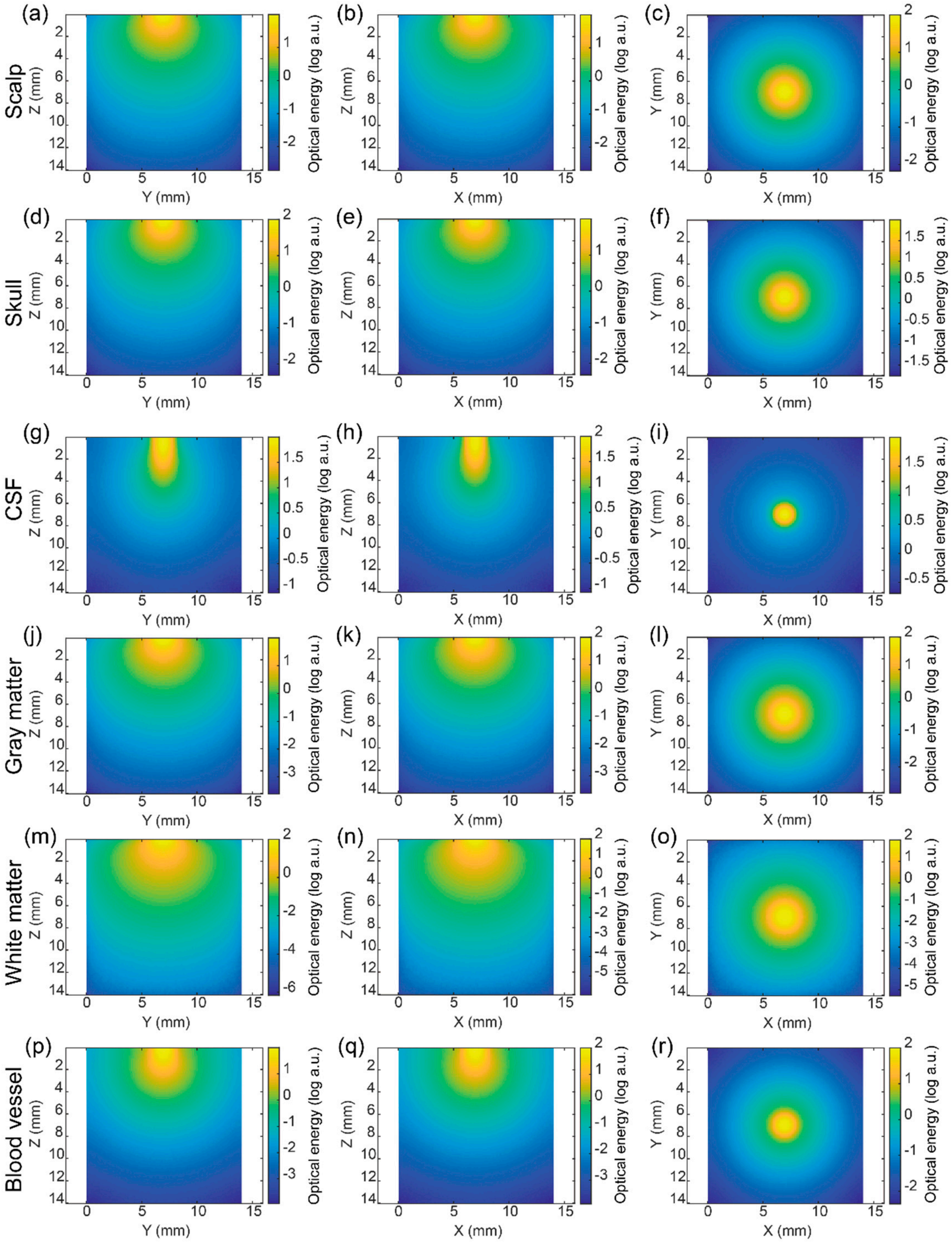

Figure S6. Optical energy distribution of single-layer brain tissues under the illumination of a collimated Gaussian source. **(a-c)** Scalp, **(d-f)** skull, **(g-i)** CSF, **(j-l)** gray matter, **(m-o)** white matter, and **(p-r)** blood vessel; **(a, d, g, j, and m)** on the YZ plane (x = 7 mm), **(b, e, h, k, and n)** on the XZ plane (y = 7 mm), **(c, f, i, l, and r)** the XY plane (z = 0.1 mm), respectively.

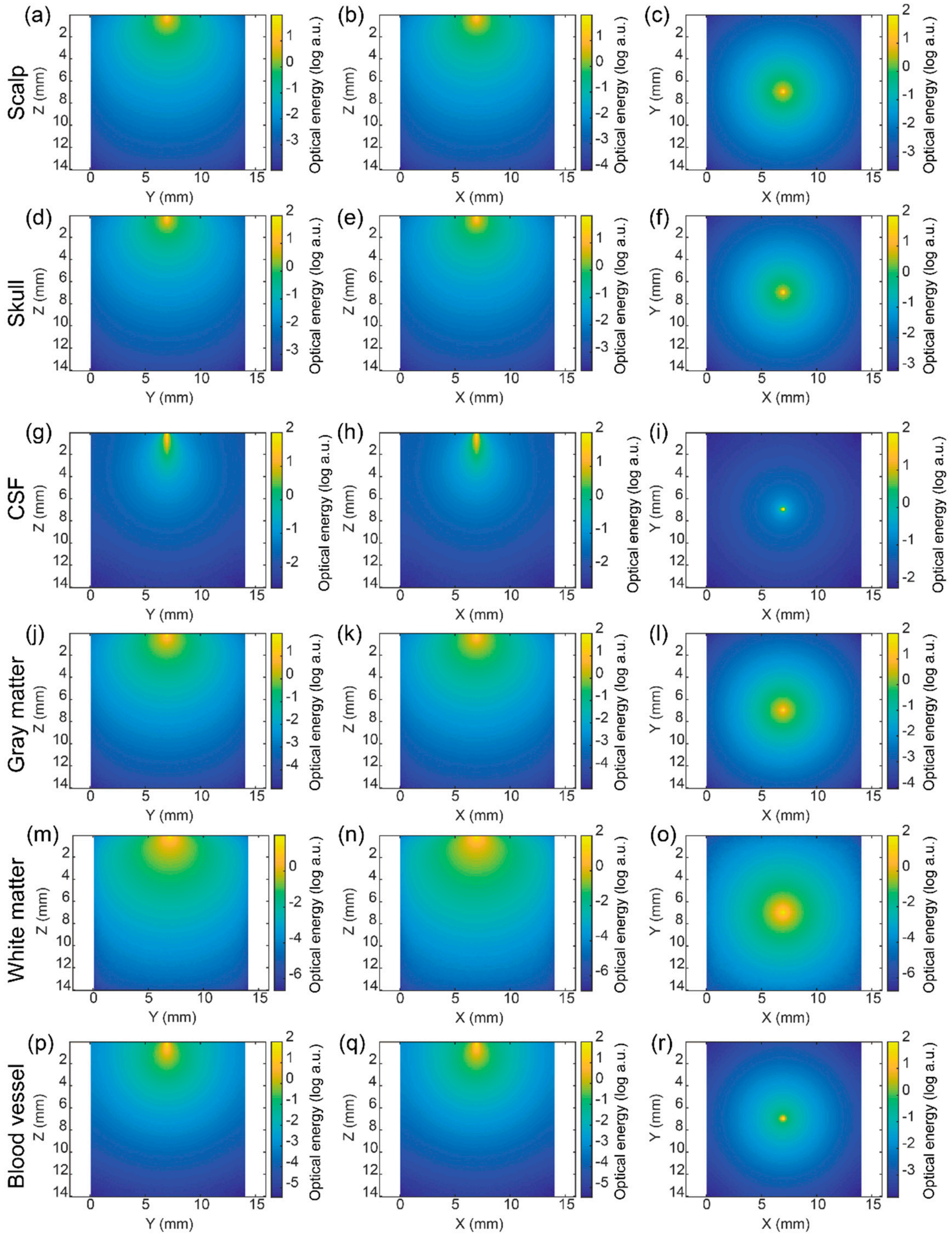

Figure S7. Optical energy distribution of single-layer brain tissues under the illumination of an angular Gaussian source. **(a-c)** Scalp, **(d-f)** skull, **(g-i)** CSF, **(j-l)** gray matter, **(m-o)** white matter, and **(p-r)** blood vessel; **(a, d, g, j, and m)** on the YZ plane (x = 7 mm), **(b, e, h, k, and n)** on the XZ plane (y = 7 mm), **(c, f, i, l, and r)** the XY plane (z = 0.1 mm), respectively.

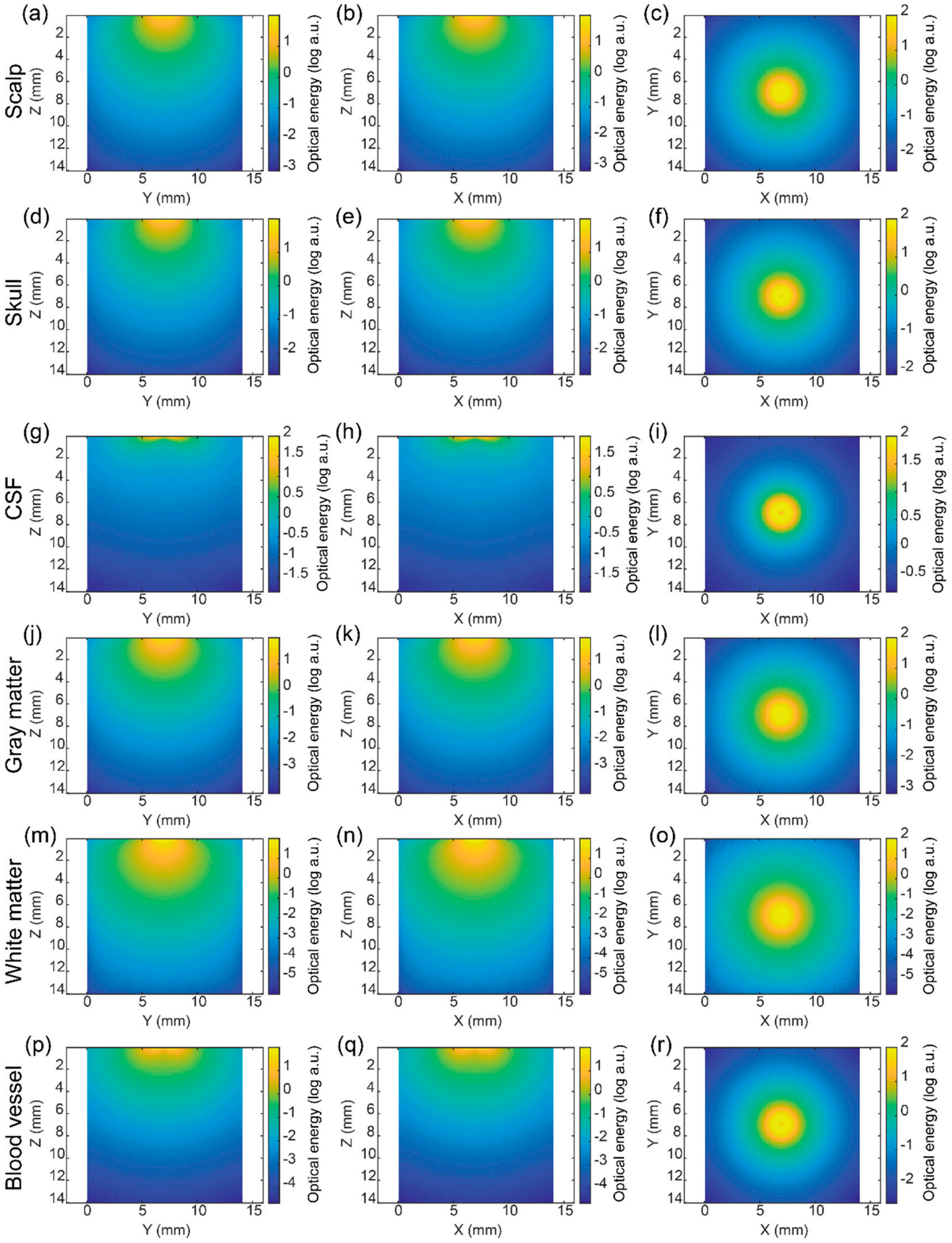

Figure S8. Optical energy distribution of single-layer brain tissues under the illumination of a hyperboloid Gaussian source. **(a-c)** Scalp, **(d-f)** skull, **(g-i)** CSF, **(j-l)** gray matter, **(m-o)** white matter, and **(p-r)** blood vessel; **(a, d, g, j, and m)** on the YZ plane (x = 7 mm), **(b, e, h, k, and n)** on the XZ plane (y = 7 mm), **(c, f, i, l, and r)** the XY plane (z = 0.1 mm), respectively.

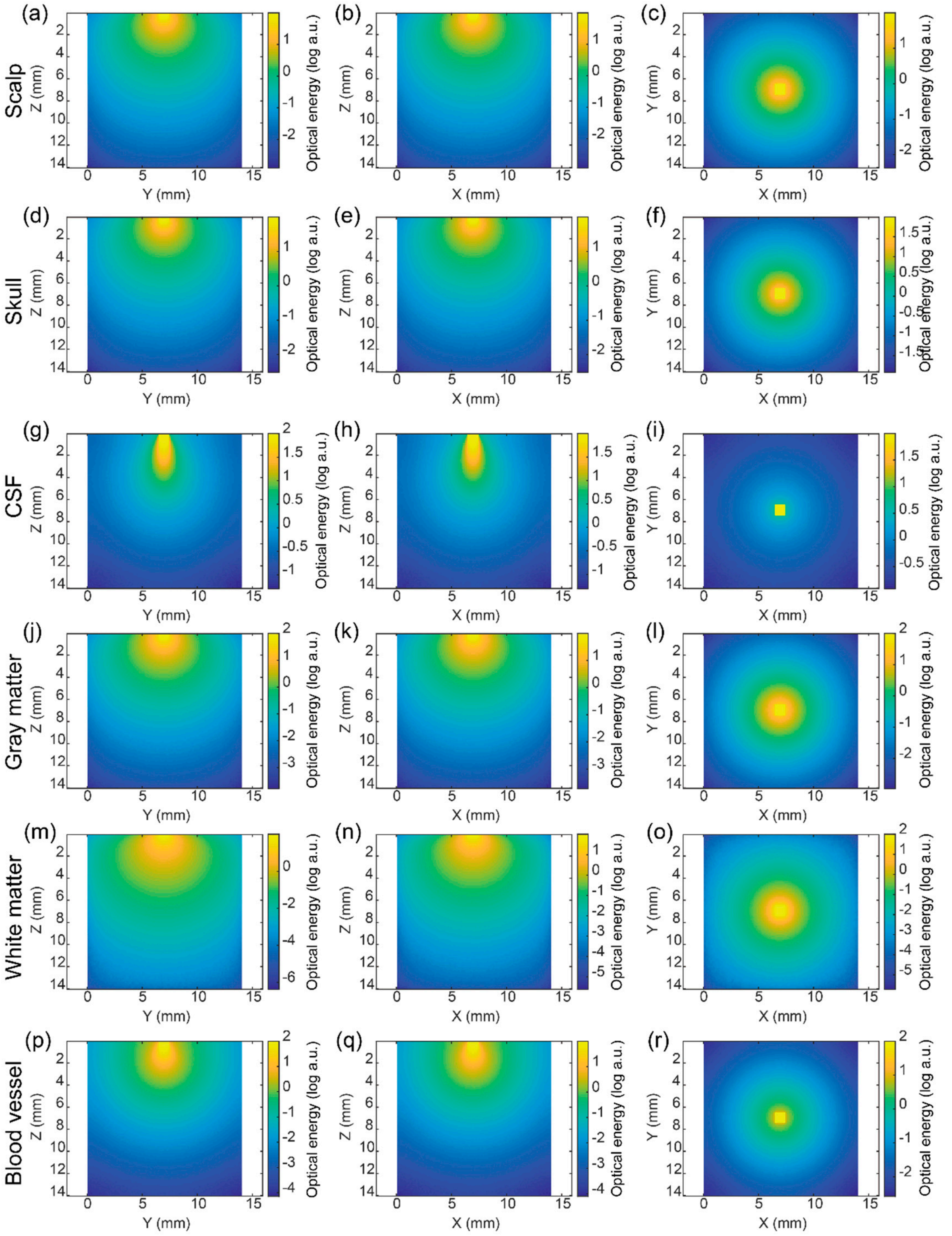

Figure S9. Optical energy distribution of single-layer brain tissues under the illumination of a planar source. **(a-c)** Scalp, **(d-f)** skull, **(g-i)** CSF, **(j-l)** gray matter, **(m-o)** white matter, and **(p-r)** blood vessel; **(a, d, g, j, and m)** on the YZ plane (x = 7 mm), **(b, e, h, k, and n)** on the XZ plane (y = 7 mm), **(c, f, i, l, and r)** the XY plane (z = 0.1 mm), respectively.

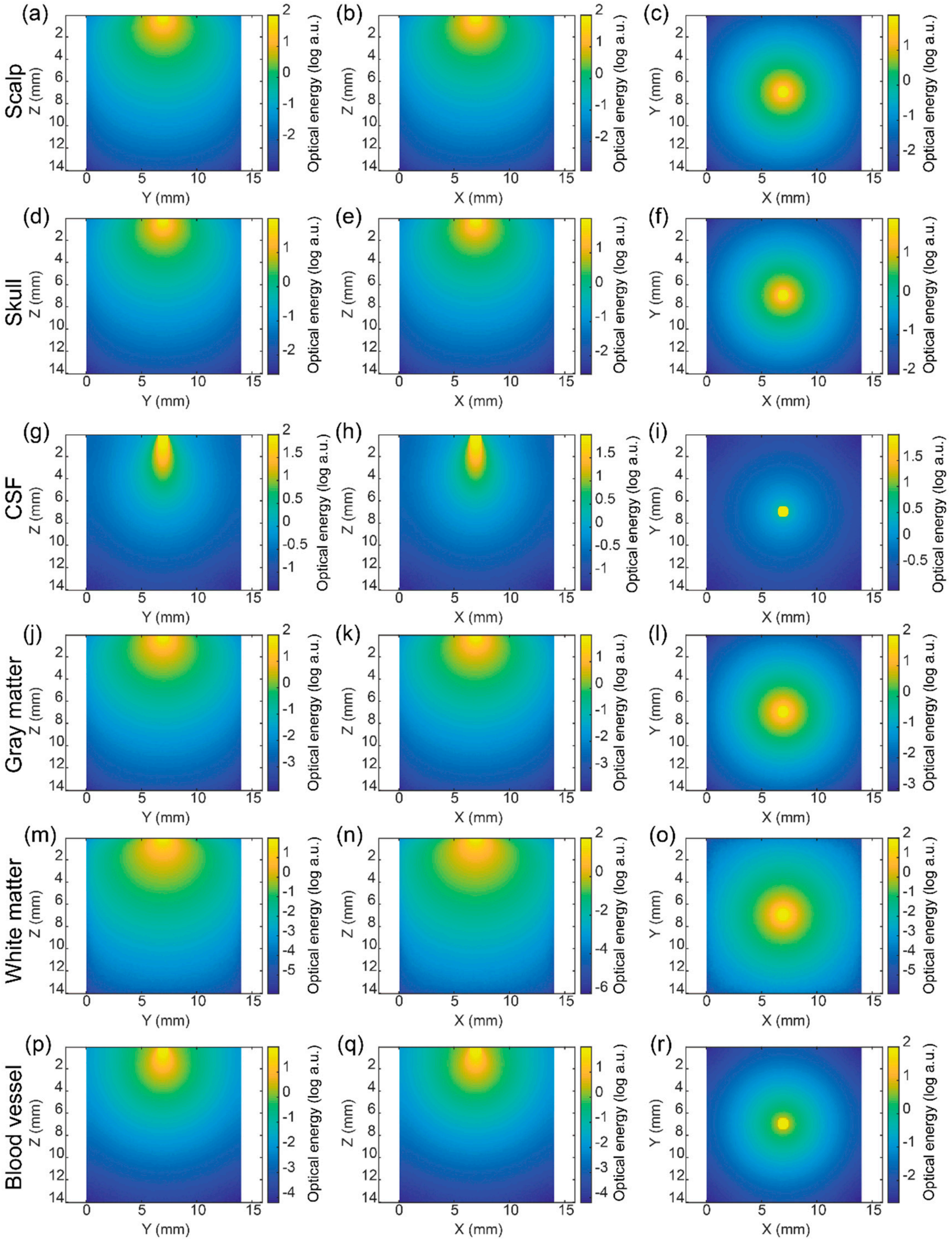

Figure S10. Optical energy distribution of single-layer brain tissues under the illumination of a disk source. **(a-c)** Scalp, **(d-f)** skull, **(g-i)** CSF, **(j-l)** gray matter, **(m-o)** white matter, and **(p-r)** blood vessel; **(a, d, g, j, and m)** on the YZ plane (x = 7 mm), **(b, e, h, k, and n)** on the XZ plane (y = 7 mm), **(c, f, i, l, and r)** the XY plane (z = 0.1 mm), respectively.

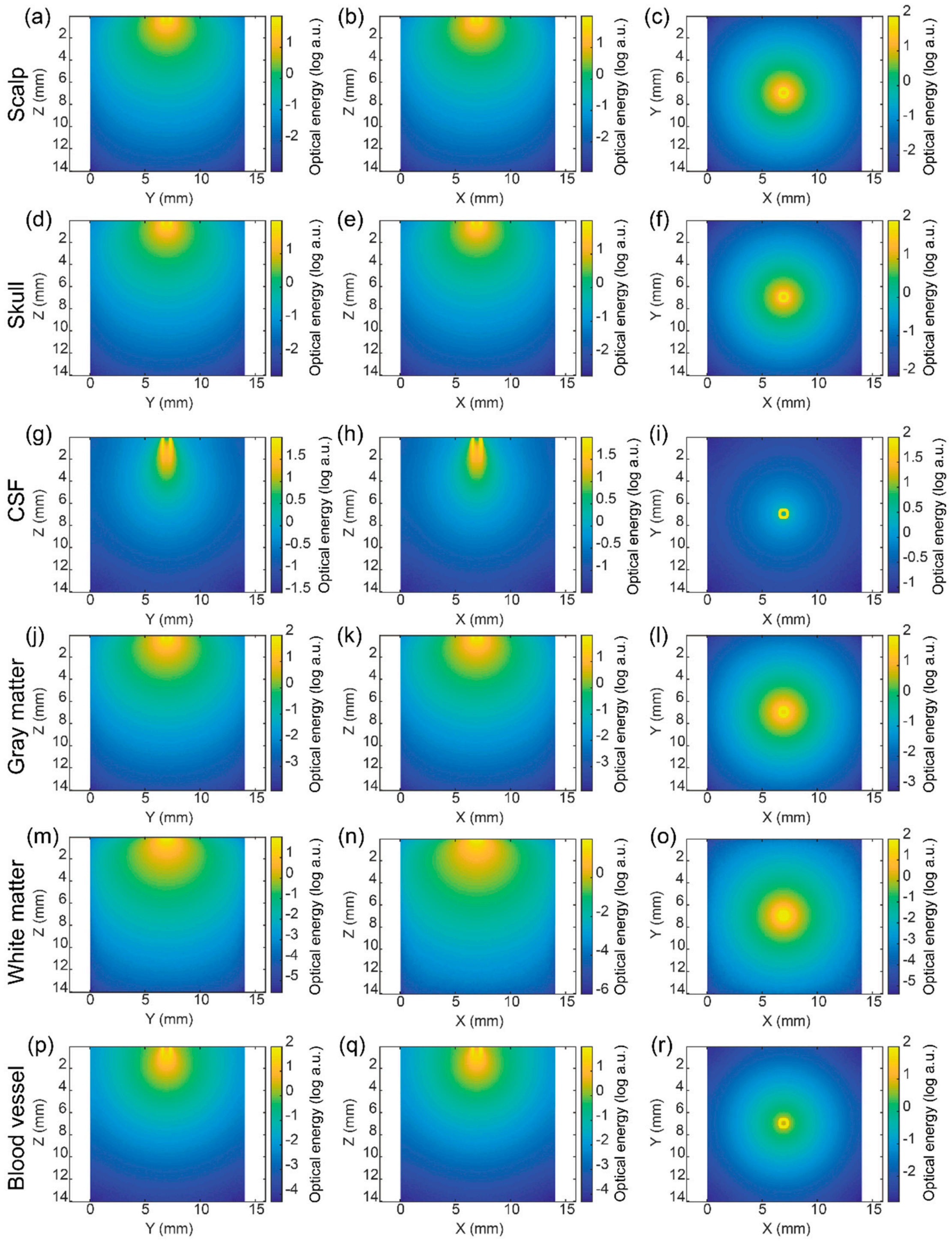

Figure S11. Optical energy distribution of single-layer brain tissues under the illumination of a ring source. **(a-c)** Scalp, **(d-f)** skull, **(g-i)** CSF, **(j-l)** gray matter, **(m-o)** white matter, and **(p-r)** blood vessel; **(a, d, g, j, and m)** on the YZ plane ($x = 7$ mm), **(b, e, h, k, and n)** on the XZ plane ($y = 7$ mm), **(c, f, i, l, and r)** the XY plane ($z = 0.1$ mm), respectively.

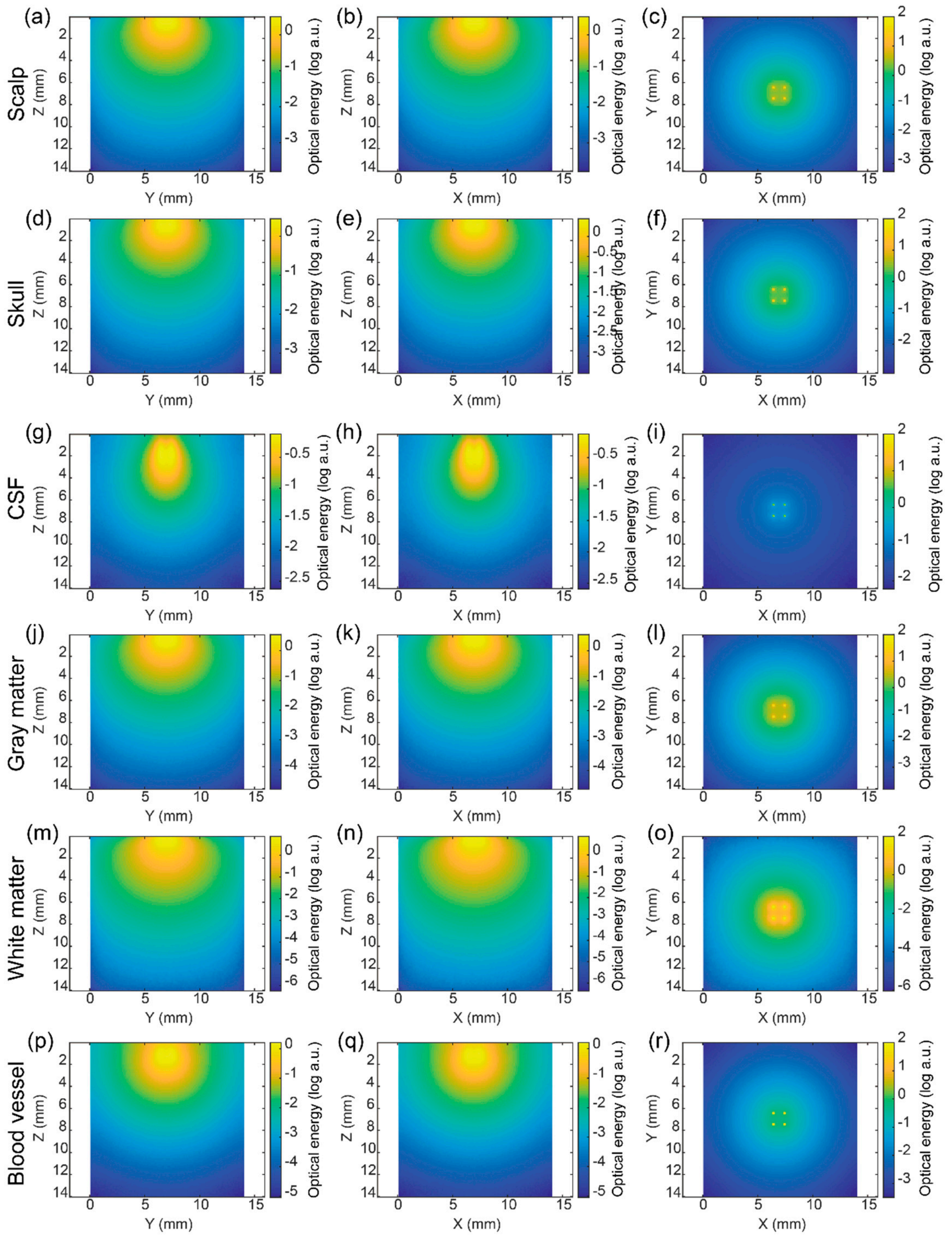

Figure S12. Optical energy distribution of single-layer brain tissues under the illumination of a pencil array source. **(a-c)** Scalp, **(d-f)** skull, **(g-i)** CSF, **(j-l)** gray matter, **(m-o)** white matter, and **(p-r)** blood vessel; **(a, d, g, j, and m)** on the YZ plane (x = 7 mm), **(b, e, h, k, and n)** on the XZ plane (y = 7 mm), **(c, f, i, l, and r)** the XY plane (z = 0.1 mm), respectively.

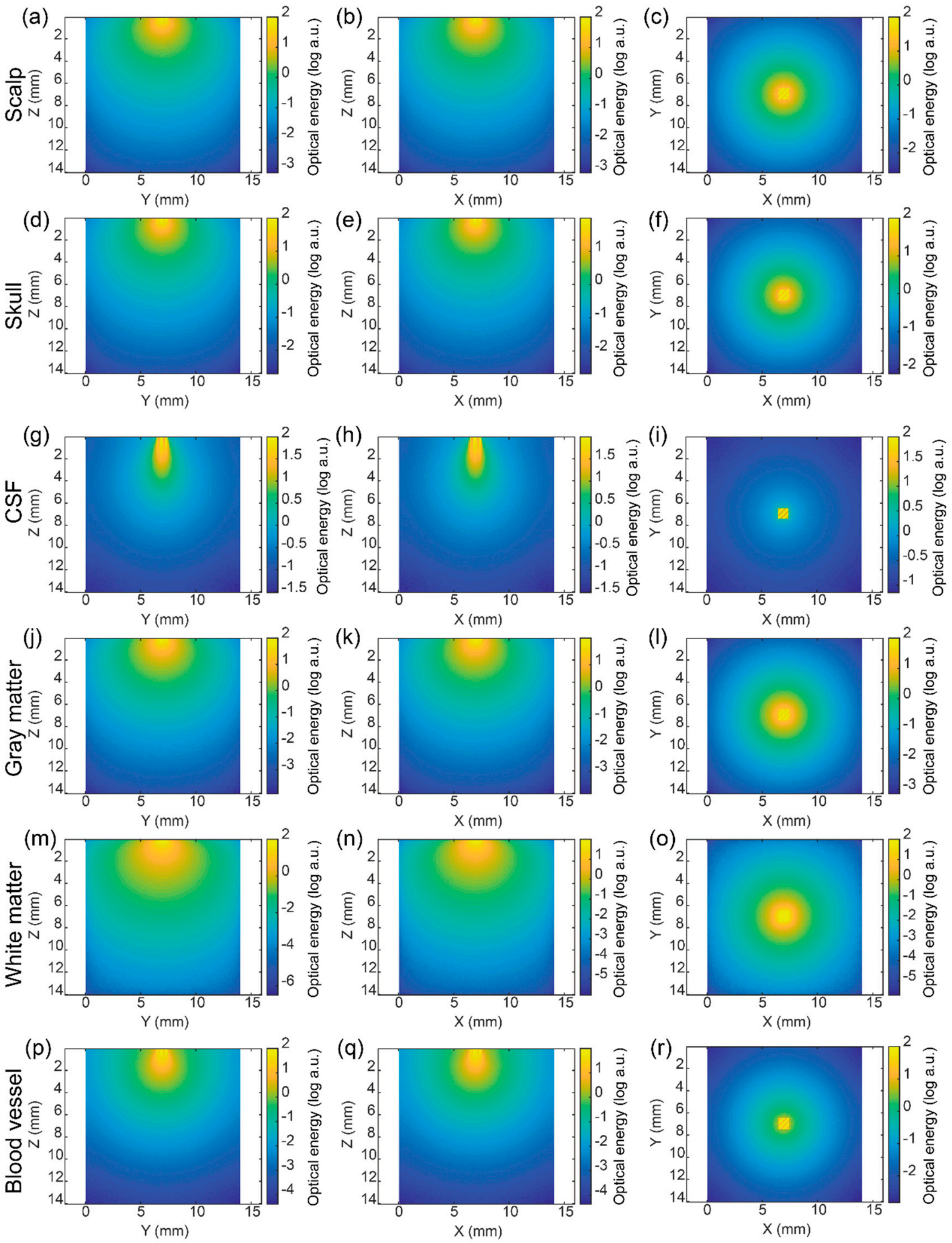

Figure S13. Optical energy distribution of single-layer brain tissues under the illumination of a spatial frequency Fourier source. **(a-c)** Scalp, **(d-f)** skull, **(g-i)** CSF, **(j-l)** gray matter, **(m-o)** white matter, and **(p-r)** blood vessel; **(a, d, g, j, and m)** on the YZ plane (x = 7 mm), **(b, e, h, k, and n)** on the XZ plane (y = 7 mm), **(c, f, i, l, and r)** the XY plane (z = 0.1 mm), respectively.

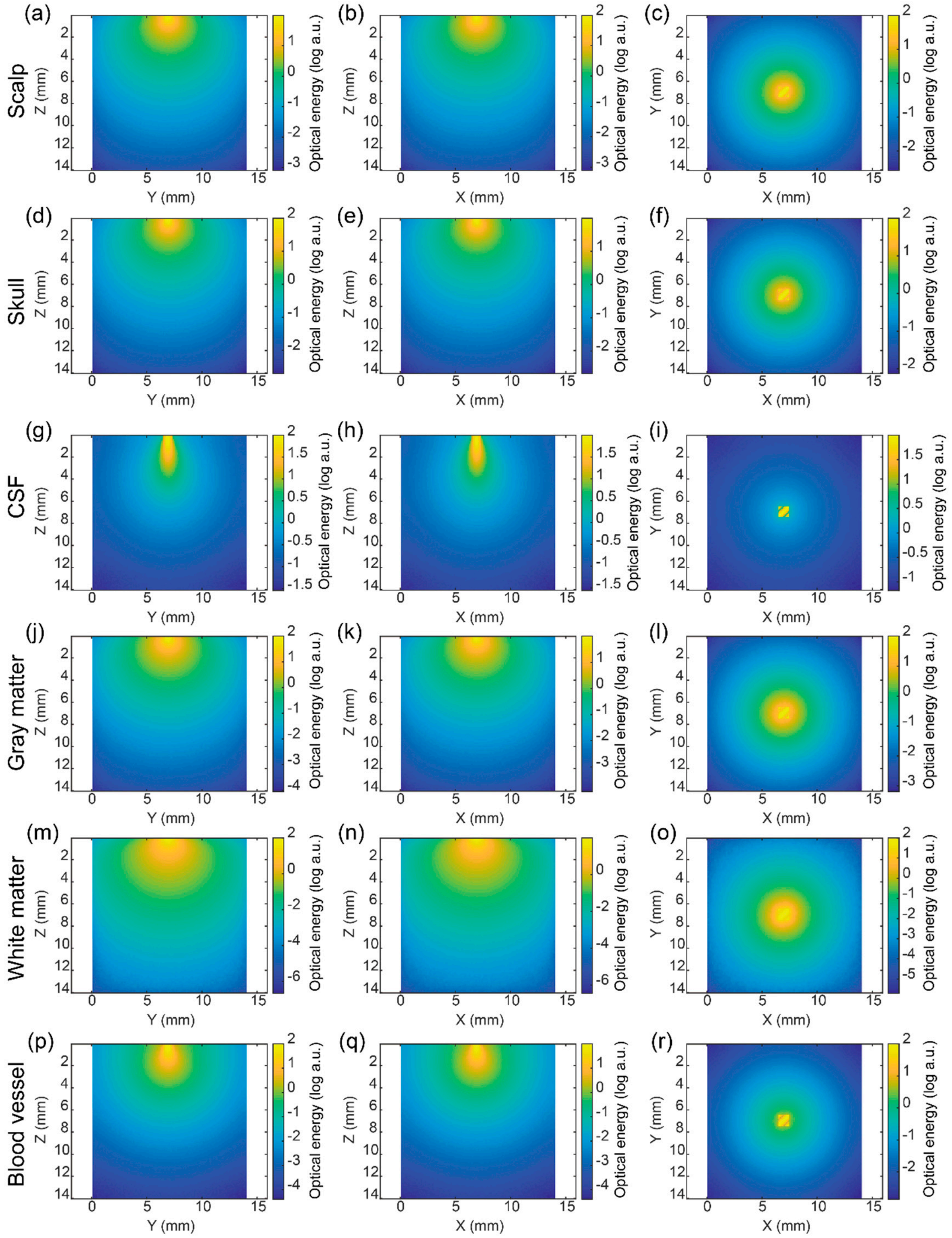

Figure S14. Optical energy distribution of single-layer brain tissues under the illumination of a 1D Fourier source. **(a-c)** Scalp, **(d-f)** skull, **(g-i)** CSF, **(j-l)** gray matter, **(m-o)** white matter, and **(p-r)** blood vessel; **(a, d, g, j, and m)** on the YZ plane (x = 7 mm), **(b, e, h, k, and n)** on the XZ plane (y = 7 mm), **(c, f, i, l, and r)** the XY plane (z = 0.1 mm), respectively.

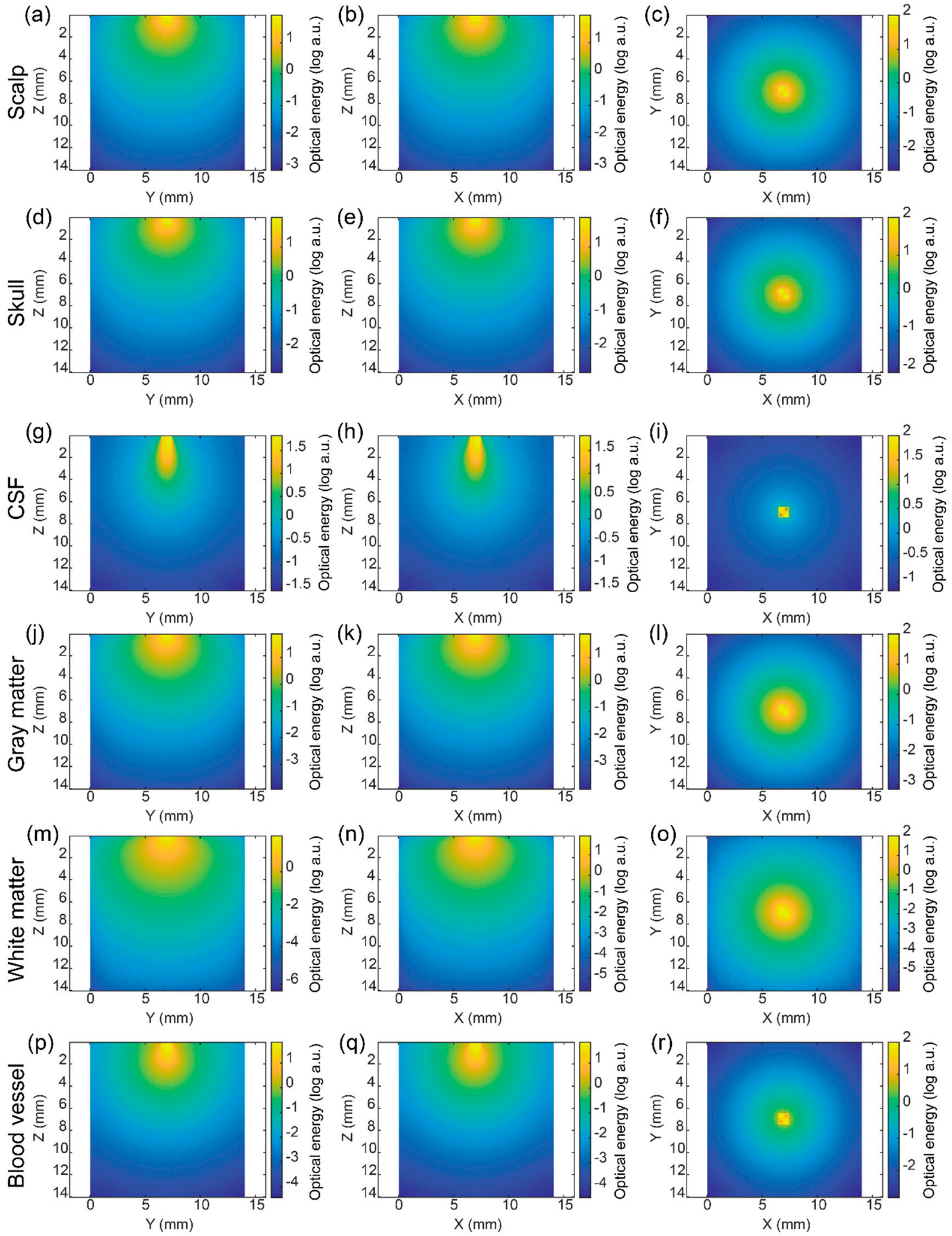

Figure S15. Optical energy distribution of single-layer brain tissues under the illumination of a 2D Fourier source. **(a-c)** Scalp, **(d-f)** skull, **(g-i)** CSF, **(j-l)** gray matter, **(m-o)** white matter, and **(p-r)** blood vessel; **(a, d, g, j, and m)** on the YZ plane (x = 7 mm), **(b, e, h, k, and n)** on the XZ plane (y = 7 mm), **(c, f, i, l, and r)** the XY plane (z = 0.1 mm), respectively.
